# Ill Results of Operative Measures in Cases of Injuries to the Brain

**Published:** 1846-10

**Authors:** B. Brodie


*Ill results of operative measures in cases of Injuries to the Brain.* By Sir B. Brodie.—There is only a small proportion of recoveries among a great number of deaths from these accidents (wounds of the brain.) I was once at the trouble of looking over all the cases of this kind which I could find recorded among my own manuscript notes, and in what might be regarded as standard books belonging to this part of surgery. I constructed a table, which represented, in every case, the kind of wound, the treatment employed by the surgeon as far as operations were concerned, and the results which followed: and it was curious to observe how large a proportion of the recoveries occurred in those cases in which the surgeon either avoided an operation altogether, or confined himself to the removal of some loose and detached pieces of bone. You may well suppose that a person who has a musket-ball lodged in the brain, is in a very serious condition; nevertheless, it appears that it is safer to allow it to remain than to endeavour to extract it.—*Sir B. Brodie's Lectures.*

